# Assessment of the Value of Rescreening for Syphilis in the Third Trimester of Pregnancy

**DOI:** 10.1155/IDOG/2006/56504

**Published:** 2006-05-30

**Authors:** Rodney K. Edwards, Margaret Bennett, Carrie Langstraat, Daina Greene

**Affiliations:** Department of Obstetrics and Gynecology, College of Medicine, University of Florida, Gainesville, FL 32610-0294, USA

## Abstract

*Objectives*. Our aim is evaluating the need for repeating tests for syphilis on pregnant women in the third trimester. *Study design.* A single-center retrospective cohort study was performed on all women delivering 7/03–6/04. *Results*. During the study interval, 2244 women delivered at our hospital. Of those women having available records and attending at least one prenatal visit, 1940 (98.9%) were screened for syphilis at the first prenatal visit. Of the 1627 women beginning prenatal care prior to 27 weeks and delivering after 32 weeks, 1377 (84.6%) were rescreened in the third trimester. No cases of syphilis were identified with either the initial (upper limit of 95% CI 0.24%) or repeat (upper limit of 95% CI 0.34%) screening. *Conclusions*. In our obstetric population, syphilis is so uncommon that mandated prenatal screening on more than one occasion seems unjustified and laws requiring repeated screening should be reevaluated.

## INTRODUCTION

The Centers for Disease Control and Prevention recommend screening all pregnant women for *Chlamydia trachomatis* infection and syphilis at the first prenatal visit. Repeated screening for these infections during the third trimester is recommended for those women at increased risk for contracting these infections. Screening for gonorrhea once or twice during pregnancy is recommended for those women at risk [1].

Most states have laws requiring that women undergo screening for syphilis at least once during pregnancy. Nine states, including Florida, have statutory requirements that pregnant women undergo screening for syphilis both at the first visit and again in the third trimester [2]. At least in Florida, there is no similar law requiring prenatal screening either for gonorrhea or *Chlamydia trachomatis* infection.

Screening for sexually transmitted diseases (STD) during pregnancy has been advocated as being cost-effective as long as the prevalence exceeds 1% [3]. The reported prevalence of gonorrhea during pregnancy varies widely, with rates varying between 0 and 10%, depending on the risk status of patients [4–6]. The prevalence of *Chlamydia trachomatis* infection during pregnancy is higher than that of gonorrhea [7, 8]. Syphilis is the least common of the sexually transmitted infections that are endemic to the United States. In 2003, cases of primary and secondary syphilis occurred at a rate of 2.5 cases per 100 000 population [9]. Since reaching a nadir in 1999, the number of cases has increased slightly over the past few years. However, this increase has been confined to men who have sex with men [9].

We sought to evaluate the prevalence of syphilis in the obstetric population delivering at Shands Hospital at the University of Florida. By doing so, we wanted to evaluate the utility of repeating screening tests for syphilis during the third trimester.

## MATERIAL AND METHODS

We performed a historical cohort analysis of all women delivering at Shands Hospital at the University of Florida from 1 July 2003 to 30 June 2004. For consideration of screening for syphilis at the first prenatal visit, all women who attended at least one prenatal visit were included. For consideration of the utility of repeating the screening in the third trimester, women were included in the cohort if they began prenatal care prior to 27-weeks gestation and delivered after 32 weeks, so as to allow a minimum of 6 weeks between first-visit testing and delivery. Screening for syphilis utilized nontreponemal tests of serum samples confirmed with treponemal tests. Screening for gonorrhea and *Chlamydia trachomatis* infection used a combined test of DNA probes from cervical swab specimens. The exact types of tests and manufacturers varied, depending on the payer-specified laboratory for specific patients. The University of Florida Health Science Center Institutional Review Board approved the study.

Subjects meeting criteria for inclusion in the cohort were identified from the delivery log maintained in the Labor and Delivery Unit at Shands Hospital at the University of Florida and from the database maintained by the Division of Maternal-Fetal Medicine at the University of Florida. Medical record charts of these women were abstracted, and data were entered into a relational database (Access 2000, Microsoft Corporation, Redmond, Wash). SAS Version 9.0 (SAS Institute, Cary, NC) was utilized for statistical analysis.

Rates of screening for syphilis at the first prenatal visit and again in the third trimester were calculated. The prevalence of infection with 95% confidence intervals (CIs) (modified Wald method) was calculated. We anticipated that the inclusion of one year of delivered patients would yield a cohort of over 1000 patients undergoing repeated third-trimester screening, allowing 95% confidence intervals of less than 3%. The unpaired Student's *t* test was used for continuous data. Categorical data were analyzed with the uncorrected chi-square and Fisher's exact tests as appropriate. All tests of statistical significance were two-tailed and utilized an alpha of 0.05.

## RESULTS

During the study interval, 2244 women delivered at our hospital, and 2085 charts were available for review. Of the 1962 women who had at least one prenatal care visit, the mean age was 25.8 (standard deviation (SD) = 6.3) years. Fifty seven percent of women were white, 25% were black, and 18% were of other ethnicities. Sixty two percent of women were parous, 64% were unmarried, 75% had either government-sponsored or “self-”payer status, and 18% reported a prior history of at least one sexually transmitted infection (gonorrhea, *Chlamydia*, syphilis, genital herpes, *trichomoniasis*, or HIV). The first prenatal visit for these patients occurred at a mean gestational age of 13.7 (SD = 6.9) weeks. Of the 1962 women who had at least one prenatal care visit, 1724 (87.9%) were screened for gonorrhea and *Chlamydia trachomatis* infection at the first prenatal care visit. Of these women, 19 (1.1%) had gonorrhea and 107 (6.2%) had a *Chlamydia trachomatis* infection.

Of the 1962 women who had at least one prenatal care visit, 1940 (98.9%) were screened for syphilis at the first prenatal care visit. No cases of syphilis were identified with this initial screening (95% confidence interval (CI) = 0,0.24%). Of the 1627 women beginning prenatal care prior to 27 weeks and delivering after 32 weeks, 1377 (84.6%) were screened in the third trimester. Similar to the results of screening at the first prenatal visit, no cases of syphilis were identified with third trimester screening (95% CI = 0,0.34%).

## DISCUSSION

Our study is limited by its retrospective nature. Also, these data are from a single center during a single year, and they may not be generalizable to other obstetric populations. However, the prevalence rates of gonorrhea and *Chlamydia trachomatis* infection in our population are similar to those in other high-risk clinic populations [10]. Therefore, we believe that it is unlikely that the prevalence of syphilis in our population is significantly lower than it would be in the majority of practice settings in the United States.

In this study, compliance with our state's legal requirement for screening for syphilis at the first visit was excellent (98.9%). Others have reported similarly high first-visit screening rates for syphilis and for other tests that are included in some sort of “prenatal battery” [11]. Compliance with the legal requirement for repeating screening in the third trimester was lower (84.6%). Reasons for this lower rate of screening than at the first visit likely include patient refusal and provider apathy due to the rarity of the infection.

The primary goal of screening pregnant women for syphilis is to prevent cases of congenital syphilis. Currently, the total number of cases in the United States each year is approximately 400 [9]. As with the rate of syphilis in women, the incidence of syphilis in newborns also is decreasing. Syphilis disproportionately affects those living in urban areas. Most counties in our state and others rarely report a case of primary or secondary syphilis in adults, much less a case of congenital syphilis.

Clearly, because of the untoward consequences of congenital syphilis, screening women once during pregnancy for this infection is reasonable and well justified. However, we believe that the statutory requirement, as currently exists in Florida and 8 other states, to screen all women twice during pregnancy for an infection that is as rare as syphilis currently should be reevaluated.
